# VAE-WACGAN: An Improved Data Augmentation Method Based on VAEGAN for Intrusion Detection

**DOI:** 10.3390/s24186035

**Published:** 2024-09-18

**Authors:** Wuxin Tian, Yanping Shen, Na Guo, Jing Yuan, Yanqing Yang

**Affiliations:** 1 School of Information Engineering, Institute of Disaster Prevention, Beijing 101601, China; 15102821950@163.com (W.T.); guona@cidp.edu.cn (N.G.); yuanjing@cidp.edu.cn (J.Y.); 2 College of Information Science and Engineering, Xinjiang University, Urumqi 830046, China; qing0991@163.com

**Keywords:** network security, network intrusion detection system (IDS), generative adversarial network, variational autoencoder, dataset balancing, deep learning

## Abstract

To address the class imbalance issue in network intrusion detection, which degrades performance of intrusion detection models, this paper proposes a novel generative model called VAE-WACGAN to generate minority class samples and balance the dataset. This model extends the Variational Autoencoder Generative Adversarial Network (VAEGAN) by integrating key features from the Auxiliary Classifier Generative Adversarial Network (ACGAN) and the Wasserstein Generative Adversarial Network with Gradient Penalty (WGAN-GP). These enhancements significantly improve both the quality of generated samples and the stability of the training process. By utilizing the VAE-WACGAN model to oversample anomalous data, more realistic synthetic anomalies that closely mirror the actual network traffic distribution can be generated. This approach effectively balances the network traffic dataset and enhances the overall performance of the intrusion detection model. Experimental validation was conducted using two widely utilized intrusion detection datasets, UNSW-NB15 and CIC-IDS2017. The results demonstrate that the VAE-WACGAN method effectively enhances the performance metrics of the intrusion detection model. Furthermore, the VAE-WACGAN-based intrusion detection approach surpasses several other advanced methods, underscoring its effectiveness in tackling network security challenges.

## 1. Introduction

With the frequent occurrence of cyber-attacks, network security has become a global concern. Intrusion detection systems (IDSs) [1], as core components of network security defense mechanisms, provide security protection by monitoring network traffic and system activities. Traditional IDSs rely on predefined intrusion patterns or signatures to identify known attacks and are less adaptable to new attack models. In contrast, machine learning-based IDSs, such as decision trees [2], random forests, and support vector machines [3], can automatically learn and identify attack signatures, significantly improving their ability to detect new attack models.

However, in typical network traffic datasets, the number of samples belonging to the normal class significantly exceeds that of the anomaly class [4]. This class imbalance phenomenon can cause machine learning-based IDSs to classify more samples as the majority class (normal traffic), thereby diminishing their ability to detect the minority class (anomalous traffic). This bias may lead IDSs to misclassify abnormal traffic as normal, which may result in failing to detect significant network attacks and can inflict severe damage on network security.

To address the challenge of class imbalance in network intrusion detection, this paper proposes an advanced generative model, VAE-WACGAN, to generate minority class samples and balance the dataset. The model builds upon the VAEGAN [5] framework by integrating key features from ACGAN [6] and WGAN-GP [7], significantly improving both the quality of generated samples and the stability of the training process. Firstly, conditional vectors are introduced to allow controlled generation of samples from specific categories, solving the issue of uncontrolled class generation in VAEGAN. Secondly, inspired by ACGAN, an auxiliary classifier is integrated into the discriminator to ensure that the samples produced by the decoder are not only highly similar to real samples but also accurately classified by the auxiliary classifier, thereby enhancing the quality of the generated samples. Lastly, the Wasserstein distance and gradient penalty from WGAN-GP are used to optimize the loss function of VAEGAN, addressing issues of training instability and poor convergence.

The class-balancing method based on the VAE-WACGAN first isolates the minority class anomaly samples from the network traffic dataset. Then, the VAE-WACGAN model learns the data distribution characteristics of these anomaly samples and generates new, diverse anomaly samples based on these characteristics. Finally, these newly generated anomaly samples are incorporated into the original dataset, enriching and balancing it.

This paper makes the following contributions:

1. This paper introduces a novel Generative Adversarial Network model named VAE-WACGAN. This model enhances the VAEGAN by integrating key features from ACGAN and WGAN-GP, significantly improving both the quality of generated samples and the stability of the training process.
2. To address the class imbalance issue in network intrusion detection, this paper proposes a class-balancing method based on the VAE-WACGAN. This method utilizes the VAE-WACGAN model to accurately learn the distribution characteristics of anomalous samples, thereby generating high-quality anomalous samples to balance the network traffic dataset.
3. Experiments are conducted on the UNSW-NB15 [8] and CIC-IDS2017 [9] datasets. The experimental results demonstrate that the VAE-WACGAN method effectively improves the performance metrics of intrusion detection models. Furthermore, the VAE-WACGAN-based intrusion detection approach outperforms several other advanced methods.

The rest of the paper is organized as follows. Section 2 reviews related work. Section 3 discusses the technical and theoretical foundations related to this study. Section 4 describes the proposed method in detail. Section 5 presents experimental validation of the proposed method and analyzes the results. Section 6 concludes the paper and explores potential avenues for future research.

## 2. Related Work

Traditional machine learning methods make predictions by learning salient features from the data. For example, Zhang et al. [10] proposed a network intrusion detection model based on a three-way selective random forest (IDTSRF) to address the low detection performance caused by the random selection of features in the random forest algorithm. This model combines three decision branches with the random forest algorithm, increasing the likelihood of key features being selected and improving detection efficacy. Experimental results demonstrate that the model achieves high precision and recall, effectively addressing some of the limitations of traditional random forests in network intrusion detection. Li et al. [11] proposed a novel anomaly-based intrusion detection system framework, CRSF, to address the issue that manually extracted features by traditional support vector machines (SVMs) may not capture all relevant information in industrial Internet environments. The CRSF framework first utilizes Convolutional Neural Networks (CNNs) and Recurrent Neural Networks (RNNs) to automatically extract spatial and temporal features from the samples, and then it employs SVMs for efficient classification. Experimental results demonstrate that the CRSF framework exhibits superior performance in intrusion detection. Despite these advancements, traditional machine learning methods still exhibit limitations when dealing with complex nonlinear data.

Compared to traditional machine learning methods, deep learning techniques are more adept at learning and understanding complex network data features, enhancing their ability to recognize various types of attacks. For instance, Li et al. [12] proposed an intrusion detection method based on Convolutional Neural Networks (CNNs). This approach initially transforms data from the NSL-KDD dataset [13] into an image format and subsequently learns features from these graphical representations. Experimental results indicate that, in most cases, this method outperforms standard classifiers. Yin et al. [14] developed an intrusion detection method utilizing recurrent neural networks (RNNs) [15]. This method leverages the strengths of RNNs in processing time series data to identify potential attacks by learning temporal dependencies and behavioral patterns within network traffic data. Experiments conducted on the KDD Cup 99 dataset [13] demonstrate that the RNN-based intrusion detection system (RNN-IDS) excels in both binary and multiclass classification tasks compared to traditional machine learning methods. Rehman et al. [16] introduced a novel vehicle intrusion detection method. This method combines Convolutional Neural Networks (CNNs) with attention-based Gated Recurrent Units (GRUs) to detect both single and mixed intrusion attacks on the CAN bus. Experimental results reveal that this approach outperforms existing methods.

Although deep learning techniques can enhance the performance of intrusion detection systems to some extent, the class imbalance issue remains a major obstacle to the advancement of this field. Common algorithms for class balancing include Random Under-Sampling (RUS) [17], Random Oversampling (ROS) [17], the Synthetic Minority Oversampling Technique (SMOTE) [18], and the Adaptive Synthetic Sampling Method (ADASYN) [19]. For example, Mishra et al. [20] increased the number of minority class samples using the ROS algorithm to create a balanced dataset, which was then used to train a deep neural network classifier for intrusion detection. Experimental results on the KDD Cup 99 dataset show that this method achieves higher accuracy in detecting all types of attacks. Wu et al. [21] proposed a network intrusion detection algorithm that combines Enhanced Random Forest with the SMOTE algorithm. The dataset was first balanced using the K-means clustering algorithm combined with the SMOTE sampling technique. The balanced dataset was then used to train the Enhanced Random Forest algorithm. Experimental results indicate that the classification accuracy of this method on the NSL-KDD dataset was 78.47%. Chen et al. [22] introduced a network intrusion detection method that combines ADASYN with Random Forest algorithms. The dataset was first balanced using ADASYN, and the balanced dataset was then used to train a Random Forest classifier. Experimental results on the CIC-IDS2017 dataset demonstrate that this method performs well in terms of accuracy, recall, F1 score, and AUC value.

However, traditional class-balancing methods often employ relatively simple sampling strategies, which struggle to fully capture the true distribution of data with complex structures. This can lead to the generation of substantial noise, negatively impacting classifier performance. With the advancement of generative models, sampling minority class samples using these models has emerged as a novel approach to addressing class imbalance issues [23].

Generative Adversarial Networks (GANs) [24] are a type of generative model that have achieved significant results in image generation [25]. Leveraging their powerful data generation capabilities, GANs can produce high-quality attack samples for network intrusion detection, effectively addressing class imbalance and enhancing model performance [26]. Specifically, Andresini et al. [27] introduced a deep learning-based binary classification method for network traffic. This method converts network traffic into 2D images, which are then used to train GANs and CNNs for effective simulation and accurate prediction of unseen network attacks. Experimental results indicate that this method achieves higher accuracy compared to other intrusion detection architectures. Ding et al. [28] proposed a tabular data sampling method to address the class imbalance in intrusion detection. This approach involves undersampling normal samples using K-nearest Neighbors and oversampling attack samples with an improved Auxiliary Classifier GAN (ACGAN), thereby balancing the dataset. Experimental results demonstrate that this method performs excellently across accuracy, F1 score, AUC, and recall metrics. Strickland et al. [29] addressed the challenge of detecting rare network attacks in existing machine learning-based intrusion detection systems (IDSs) with a novel hybrid approach. This method first uses GANs for data oversampling and then trains a Deep Reinforcement Learning (DRL) model on the augmented data. Experimental results show that this approach significantly improves the detection capability for rare network attacks.

The Variational Autoencoder Generative Adversarial Network (VAEGAN) is a notable variant of the GAN. Compared to a traditional GAN, it combines the advantages of Variational Autoencoders (VAEs) [30] and GANs, providing a more stable training process and higher-quality sample generation. Originally applied in image generation to produce clearer images, VAEGAN’s exceptional performance has led to its gradual adoption in the field of data augmentation for generating high-quality data. To illustrate, Ding et al. [31] proposed an improved VAEGAN oversampling method based on dual encoders to address the dataset imbalance in credit card fraud detection. Results show that oversampling with this method significantly improved the fraud detection model’s precision, F1 score, and other metrics. Li et al. [32] introduced an improved VAEGAN model to address the lack of Arterial Spin Labeling (ASL) image data in dementia imaging datasets. The modified VAEGAN effectively generates ASL images, significantly enhancing accuracy in dementia detection tasks. Wang et al. [33] proposed a Conditional Variational Autoencoder Generative Adversarial Network (CVAE-GAN) to generate fault samples and balance datasets in planetary gearbox fault diagnosis. Results indicate that the CVAE-GAN effectively generates fault samples under varying operating conditions, improving fault diagnosis performance. Tang et al. [34] addressed the performance degradation in interference recognition with small sample sets by combining ACGAN and VAE ideas, proposing an Auxiliary Classifier Variational Autoencoder Generative Adversarial Network (AC-VAEGAN) to expand datasets and improve interference recognition accuracy. Experimental results demonstrate that this method achieves higher recognition rates across five different interference types. He et al. [35] improved the VAEGAN to address the class imbalance in network intrusion detection, introducing a new network, CWVAEGAN, to generate minority class samples. They developed an intrusion detection system based on the CWVAEGAN and a one-dimensional Convolutional Neural Network (1DCNN). Experimental results show that the CWVAEGAN-1DCNN outperforms other advanced methods.

Despite the success of VAEGAN in other areas, its application to addressing class imbalance in network intrusion detection remains relatively unexplored. Additionally, VAEGAN faces challenges in terms of training stability and the quality of generated samples. To tackle these limitations, this paper proposes VAE-WACGAN, which combines the key features of WGAN-GP and ACGAN into VAEGAN. This new model improves training stability and sample quality by using the Wasserstein distance and gradient penalty from WGAN-GP, and an auxiliary classifier from ACGAN. By oversampling the minority class using VAE-WACGAN, the generated anomaly samples better align with actual network traffic, balancing the dataset and enhancing the overall performance of the intrusion detection model.

## 3. Background

### 3.1. Variational Autoencoder (VAE)

**Figure 1:**
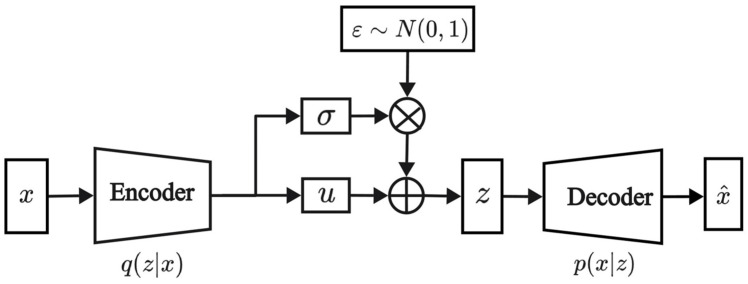
Model structure of VAE.

VAE [30] is a generative model composed of an encoder and a decoder. The encoder transforms input data into latent variables, while the decoder reconstructs these latent variables into samples that resemble the input data. This method enables VAE to learn the complex distribution of the input data and generate similar samples. The VAE structure is depicted in Figure 1, and the loss function is outlined below.

(1) $$L_{\mathrm{VAE}}=L_{\mathrm{like}}^{\mathrm{pixel}}+L_{\mathrm{prior}}=-E_{q(z|x)}\left[\log p(x|z)\right]+D_{\mathrm{KL}}\left(q(z|x)∥p(z)\right)$$

In Equation (1), x represents the input data, and z represents the latent variables. The term $-E_{q(z|x)}\left[\log p(x|z)\right]$ represents the reconstruction error, which measures the discrepancy between the input and reconstructed data. $D_{\mathrm{KL}}\left(q(z|x)∥p(z)\right)$ denotes the Kullback–Leibler divergence, which measures the difference between the posterior distribution q(z|x) and the prior distribution p(z).

### 3.2. Generative Adversarial Networks (GANs) and Their Variants

#### 3.2.1. Generative Adversarial Networks (GANs)

**Figure 2:**
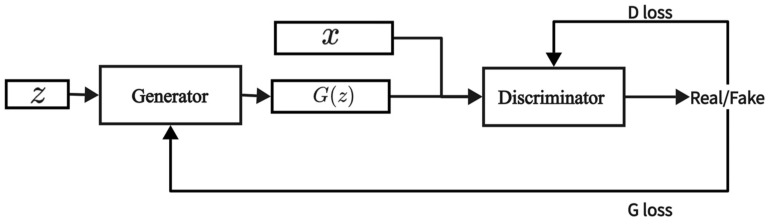
Model structure of GAN.

A GAN [24] is also a type of generative model, consisting of two main components: a generator and a discriminator. The generator attempts to create realistic data to deceive the discriminator, while the discriminator strives to distinguish between the real data and the fake data produced by the generator. The generator and discriminator engage in a continuous adversarial process, which trains the generator to produce high-quality data. The structure of the GAN is illustrated in Figure 2, and the objective function is presented below.

(2) $$L(G,D)=\underset{G}{\min}\;\underset{D}{\max}E_{x\sim p_{\mathrm{data}}}[\log D(x)]+E_{z\sim p_{z}}[\log(1-D(G(z)))]$$

In Equation (2), x represents the real data, z represents the random noise vector, D(x) denotes the discriminator’s score for real data, and D(G(z)) denotes the discriminator’s score for fake data. The distribution of real data is represented by $p_{\mathrm{data}}$, and the distribution of the random noise vector is represented by $p_{z}$.

#### 3.2.2. Variational Autoencoder Generative Adversarial Network (VAEGAN)

VAEGAN [5] is a deep learning model that integrates Variational Autoencoders (VAEs) and Generative Adversarial Networks (GANs), aiming to enhance the quality of generated samples by combining the strengths of both. In VAEGAN, the encoder from the VAE maps input data to a latent space, while the decoder acts as the generator in the GAN, creating new data samples. Simultaneously, the discriminator is trained to distinguish between generated and real samples. VAEGAN combines the reconstruction loss of the VAE with the adversarial loss of the GAN, resulting in samples that are both realistic and retain the underlying structure of the data, effectively improving the quality of generated samples. The loss function of VAEGAN comprises two components: the VAE loss function $L_{\mathrm{VAE}}$ and the GAN loss function $L_{\mathrm{GAN}}$, presented as follows:

(3) $$L=L_{\mathrm{VAE}}+L_{\mathrm{GAN}}$$

#### 3.2.3. Auxiliary Classifier Generative Adversarial Network (ACGAN)

ACGAN [6] is an enhanced version of GAN, incorporating an auxiliary classifier into the traditional GAN framework. The generator of ACGAN not only tries to produce fake samples that deceive the discriminator but also ensures that these generated samples adhere to additional categorical constraints. The loss function of ACGAN consists of two parts: the adversarial loss $L_{S}$ and the classification loss $L_{C}$. The training objective of the discriminator is to minimize $L_{S}+L_{C}$, while the training objective of the generator is to minimize $L_{C}-L_{S}$.

(4) $$L_{S}=-E[\log{P(S=\mathrm{real}|X}_{\mathrm{real}})]-E[\log{P(S=\mathrm{fake}|X}_{\mathrm{fake}})]$$

(5) $$L_{C}=-E[\log{P(C=c|X}_{\mathrm{real}})]-E[\log{P(C=c|X}_{\mathrm{fake}})]$$

where P(S|X) denotes the probability that the discriminator D discriminates the sample X as real or fake data, with S taking the values “real” or “fake”. P(C|X) represents the probability that the discriminator D discriminates the sample X belonging to a specific class, with C corresponding to the possible class labels.

#### 3.2.4. Wasserstein Generative Adversarial Network with Gradient Penalty Term

WGAN-GP [7] is an improved version of WGAN. WGAN (Wasserstein Generative Adversarial Network) [36] utilizes the Wasserstein distance to measure the distribution difference between generated and real samples, addressing the training instability issues caused by the use of Jensen–Shannon divergence in traditional GANs. WGAN-GP introduces a gradient penalty to ensure the discriminator’s gradients meet the K-Lipschitz continuity condition, further stabilizing the training process and preventing issues such as gradient explosion or vanishing. The loss function consists of two components: the discriminator’s loss function $L_{D}$ and the generator’s loss function $L_{G}$.

(6) $$L_{D}=E[D(x)]-E[D(G(z))]+\lambda \cdot E\left[{\left(\|\nabla_{\hat{x}}D(\hat{x}){\|}_{2}-K\right)}^{2}\right]$$

(7) $$L_{G}=-E[D(G(z))]$$

where λ is the weighting factor for the gradient penalty, and $\nabla_{\hat{x}}D(\hat{x})$ represents the gradient of the discriminator’s function at $\hat{x}$, which is a random linear interpolation between real and generated samples. K is a constraint typically set to 1, corresponding to the 1-Lipschitz constraint.

## 4. Methods

In this section, a detailed introduction is provided for the VAE-WACGAN model proposed in this paper, and an intrusion detection system framework based on VAE-WACGAN is established for addressing class imbalance in network intrusion detection.

### 4.1. VAE-WACGAN

**Figure 3:**
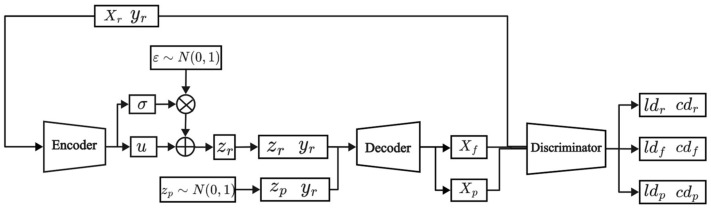
Model structure of VAE-WACGAN.

The model structure of the VAE-WACGAN is illustrated in Figure 3, where the model comprises three components: an encoder, a decoder, and a discriminator. The discriminator integrates both a traditional discriminator and an auxiliary classifier. The model structure, loss function, and training process of VAE-WACGAN will be sequentially introduced in Section 4.1.

#### 4.1.1. Model Structure

(1) Encoder

The detailed network structure of the encoder is presented in Table 1. The encoder receives the original feature vector $X_{r}$ and the conditional vector $y_{r}$ as inputs. Initially, the input conditional vector $y_{r}$ is converted into an embedded representation through an embedding layer and then merged with the original feature vector $X_{r}$ to form a composite feature vector. Subsequently, this composite feature vector passes through two convolutional layers for feature extraction. The extracted feature vector is then flattened and transformed through a fully connected layer in terms of feature dimensions. Then, two separate fully connected layers are used to calculate the mean u and variance $\sigma^{2}$ in the latent space, which together define the Gaussian distribution of the latent variable. Finally, $z_{r}$ is sampled from the defined Gaussian distribution using the reparameterization trick, with a dimension of 128.

In Table 1, Table 2 and Table 3, “feature_num” indicates the number of features per sample (i.e., the dimension of the sample), “classes” represents the total number of categories to which the samples belong, “Filters” refers to the number of output channels of the 1-dimensional convolutional layer, “Kernel Size” denotes the size of the convolutional kernel, “Output shape” shows the dimensions of the sample after passing through the layer, and “Activation” specifies the activation function used after the layer. Additionally, the stride of the 1-dimensional convolutional layer is set to 1, and padding is set to 0.

(2) Decoder

The detailed network structure of the decoder is outlined in Table 2. The decoder functions to generate reconstructed samples, $X_{f}$, or entirely new samples, $X_{p}$, based on the given latent variable $z_{r}$ (or the equivalent random noise vector $z_{p}$) and the conditional vector $y_{r}$.

Initially, the decoder converts the conditional vector $y_{r}$ into an embedding vector via an embedding layer, which is then merged with the latent variable $z_{r}$ (or the random noise vector $z_{p}$), resulting in a composite feature vector. Subsequently, this composite feature vector undergoes a dimension transformation through a fully connected layer to accommodate the input requirements of subsequent layers. Then, the dimensionally transformed vector is restructured into multidimensional data via an Unflatten operation, which restores the spatial structure for the transposed convolution operation. Finally, the data are upsampled through three transpose convolution layers, progressively increasing their dimensions to generate the final reconstructed samples, $X_{f}$, or the newly created samples, $X_{p}$.

(3) Discriminator

The detailed network structure of the discriminator is shown in Table 3. The discriminator receives raw data $X_{r}$, reconstructed data $X_{f}$, or newly generated data $X_{p}$ as inputs. The input data first pass through two convolutional layers for feature extraction. Subsequently, the feature extracted vector is flattened and then transformed through two fully connected layers for feature dimension conversion. Next, the network splits into two main branches: the upper branch assesses the authenticity of the input data, outputting the authenticity score ld through a fully connected layer. The lower branch focuses on category prediction, outputting the category prediction cd through a fully connected layer.

#### 4.1.2. Loss Function

In the loss function of the VAE-WACGAN, the decoder is denoted by the symbol G. The discriminator’s outputs are represented by the symbols S and C, where S(x) indicates the discriminator’s judgment on whether the input sample x is real, and C(x) represents the prediction of the category to which sample x belongs.

(1) Encoder

The encoder’s loss function incorporates class information, thereby enhancing its ability to capture and distinguish key features of different categories in the latent space. The encoder’s loss function is defined as follows:

(8) $$L_{\mathrm{encoder}}=L_{\mathrm{prior}}+\Upsilon \cdot L_{recon}$$

(9) $$L_{\mathrm{prior}}=D_{KL}\left(q(z|x,c)∥p(z|c)\right)$$

(10) $$L_{recon}=-E_{q(z|x,c)}\left[\log p(x|z,c)\right]$$

where x represents the real sample $X_{r}$, z denotes the latent variable $z_{r}$, and c refers to the conditional vector of $X_{r}$. q(z|x,c) is the approximate posterior distribution of the latent variable z as inferred by the encoder. p(z|c) is the prior distribution of the latent variable z. p(x|z,c) represents the distribution of the data x fitted by the decoder.

$L_{\mathrm{prior}}$ represents the Kullback–Leibler Divergence (KLD) loss, which is employed to minimize the discrepancy between the encoded latent variable distribution and the prior distribution. This ensures that the latent variable distribution produced by the encoder approximates the predetermined prior distribution, thereby enhancing the generalization capability of the encoding process. $L_{\mathrm{recon}}$ denotes the reconstruction loss, which aims to minimize the difference between the original samples $X_{r}$ and the reconstructed samples $X_{f}$. This loss encourages the encoder to accurately capture the essential features of the input data, enabling the decoder to reconstruct the original input effectively. Υ is the weighting factor for the reconstruction loss term.

(2) Decoder

The decoder’s loss function has the following features: Firstly, it incorporates class information, encouraging the decoder to pay more attention to class characteristics when generating data. Secondly, it uses the Wasserstein distance to optimize the adversarial loss, significantly enhancing the stability of the training process. Lastly, it includes a classification loss component to impose class constraints on the generated samples. The decoder’s loss function is as follows:

(11) $$L_{Decoder}=L_{S}+L_{C}+\Upsilon \cdot L_{recon}$$

(12) $$L_{S}=-E[S(G(z|c))]$$

(13) $$L_{C}=-E[\log P(C(G(z|c))=c)]$$

where z denotes the random noise vector $z_{p}$, and c refers to the conditional vector of $X_{r}$. G(z|c) represents the sample generated by the decoder given the condition c and random noise z, which is the newly generated sample $X_{p}$. S(G(z|c)) is the discriminator’s score for the authenticity of the sample generated by the decoder; the higher the score, the more realistic the sample, and the lower the score, the less realistic the sample. P(C(G(z|c))=c) represents the probability that the discriminator correctly predicts the class of the sample G(z|c) generated by the decoder.

$L_{S}$ is the adversarial loss for the decoder, used to maximize the probability that the samples generated by the decoder are judged as real, thereby optimizing the decoder to produce more realistic samples. $L_{C}$ represents the classification loss for the decoder, used to maximize the probability that the samples generated by the decoder are correctly classified. This optimization ensures that the generated samples are not only realistic but also correctly classified, further enhancing the quality of the generated samples. The reconstruction loss $L_{recon}$ guides the decoder to accurately reconstruct the input data from the encoded latent space, ensuring that the generated output closely resembles the original input.

(3) Discriminator

The discriminator’s loss function uses the Wasserstein distance and gradient penalty term to optimize the adversarial loss, effectively addressing the instability and convergence issues encountered during the training of VAEGAN. Additionally, the loss function includes a classification loss component to enhance the discriminator’s ability to recognize data. The discriminator’s loss function is as follows:

(14) $$L_{D}=L_{S}+L_{C}$$

(15) $$L_{S}=-E[S(x)]+E[S(G(z|c))]+\lambda E\left[{(∥\nabla_{\hat{x}}S(\hat{x}){∥}_{2}-1)}^{2}\right]$$

(16) $$L_{C}=-E[\log P(C(x)=c)]-E[\log P(C(G(z|c))=c)]$$

where x refers to the real sample $X_{r}$, z refers to both the latent variable $z_{r}$ and the random noise vector $z_{p}$, and c is the conditional vector of $X_{r}$. $\hat{x}$ represents the randomly interpolated sample between the real sample $X_{r}$ and the generated sample $X_{p}$. G(z|c) denotes the samples generated by the decoder, which include both the reconstructed sample $X_{f}$ and the newly generated sample $X_{p}$.

In $L_{S}$, −E[S(x)]+E[S(G(z|c))] optimizes the discriminator by maximizing its score for real samples and minimizing its score for samples generated by the decoder, thereby enhancing its ability to distinguish between real and fake data. The gradient penalty term $\lambda E\left[{\left(∥\nabla_{\hat{x}}S(\hat{x}){∥}_{2}-1\right)}^{2}\right]$ ensures that the discriminator satisfies the 1-Lipschitz continuity condition, improving the stability of the training process.

In $L_{C}$, −E[logP(C(x)=c)] maximizes the probability that the real samples are correctly classified, while −E[logP(C(G(z|c))=c)] maximizes the probability that the samples generated by the decoder are correctly classified. This improves the discriminator’s ability to recognize both real and fake data.

#### 4.1.3. Training Process

During the training of the VAE-WACGAN, the model consists of two parts: the generative part (encoder and decoder) and the discriminative part (discriminator). These two components are alternately trained to continuously optimize their respective performances. The complete training process of the VAE-WACGAN is illustrated in Algorithm 1. 

In Algorithm 1, E, G, and D denote the encoder, decoder, and discriminator, respectively. $\theta_{E}$, $\theta_{G}$, and $\theta_{D}$ represent the parameters of the encoder, decoder, and discriminator. $n_{critic}$ specifies the number of times the discriminator is trained before each training of the encoder and decoder. The meanings of the remaining symbols are consistent with those in Section 4.1.2.
**Algorithm 1** Training the VAE-WACGAN model1:$\theta_{E},\theta_{G},\theta_{D}\leftarrow$ initialize network parameters2:**repeat**3:  **for** i=1
→
$n_{critic}$ **do**4:**    **$(X_{r},y_{r})\leftarrow$ random mini-batch from dataset5:**    **$z_{r}\leftarrow E(X_{r},y_{r})$6:**    **$X_{f}\leftarrow G(z_{r},y_{r})$7:**    **$z_{p}\leftarrow$ samples from prior N(0,I)8:**    **$X_{p}\leftarrow G(z_{p},y_{r})$9:**    **// Update the parameters of the Discriminator10:**    **$L_{S}\leftarrow -E[{S(X}_{r})]+E[{S(X}_{f})]+E[{S(X}_{p})]+\lambda E[{(∥\nabla_{\hat{x}}S(\hat{x}){∥}_{2}-1)}^{2}]$11:**    **$L_{C}\leftarrow -\left(E[\log{P(C(X}_{r}{)=y}_{r})]-E[\log{P(C(X}_{f}{)=y}_{r})]-E[\log{P(C(X}_{p}{)=y}_{r})]\right)$12:**    **$L_{D}\leftarrow L_{S}+L_{C}$13:**    **$\theta_{D}\leftarrow \mathrm{Adam}(\theta_{D},\nabla_{\theta_{D}}(L_{D}))$14:**  end for**15:**  **// Update the parameters of the Encoder and Decoder16:**  **$L_{prior}\leftarrow D_{KL}(q(z_{r}|X_{r},y_{r})∥p(z_{r}|y_{r}))$17:**  **$L_{recon}\leftarrow -E_{q(z_{r}|X_{r},y_{r})}\left[\log p(X_{r}|z_{r},y_{r})\right]$18:**  **$L_{E}\leftarrow L_{prior}+\Upsilon \cdot L_{recon}$19:**  **$L_{G}\leftarrow -E[{S(X}_{p})]-E[\log{P(C(X}_{p}{)=y}_{r})]+\Upsilon \cdot L_{\mathrm{recon}}$20:**  **$\theta_{E}\leftarrow \mathrm{Adam}(\theta_{E},\nabla_{\theta_{E}}(L_{E}))$21:**  **$\theta_{G}\leftarrow \mathrm{Adam}(\theta_{G},\nabla_{\theta_{G}}(L_{G}))$22:**until** D has converged to 0.5

### 4.2. IDS Based on VAE-WACGAN

**Figure 4:**
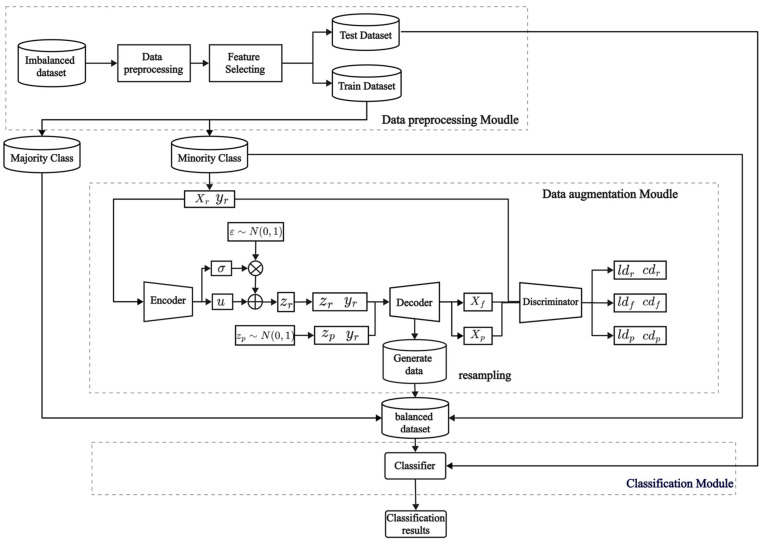
Model structure of VAE-WACGAN-IDS.

Based on the VAE-WACGAN method proposed in Section 4.1, a framework called VAE-WACGAN-IDS is constructed. This framework includes three modules: the data preprocessing module, the data augmentation (VAE-WACGAN) module, and the classification module, as shown in Figure 4. Firstly, the data preprocessing module performs preprocessing and feature selection on the raw imbalanced dataset. Secondly, the VAE-WACGAN method is applied to generate samples of the minority class, creating a balanced dataset. Lastly, the classifier is trained using the balanced dataset to develop a network intrusion detection model for addressing imbalanced network intrusion detection.

#### 4.2.1. Data Preprocessing Module

The data preprocessing module is responsible for preprocessing the raw imbalanced dataset to enhance data quality and meet the requirements for the subsequent model training. This module encompasses two main steps: data preprocessing and feature selection.

Data preprocessing includes data cleaning, feature encoding, and normalization. Data cleaning primarily deals with null values and infinity values in the dataset. Feature encoding is used to convert non-numeric features into numeric form. Normalization adjusts the scale of the data to a common range to reduce the scale differences between the features and improve the convergence speed of the algorithm.

After data preprocessing, the dataset exhibits high-dimensional sparsity, which could adversely affect the performance of subsequent models. Consequently, the Random Forest algorithm is employed to select the most relevant features. 

#### 4.2.2. Data Augmentation Module

The data augmentation module is responsible for generating samples of the minority class to balance the class distribution of the dataset. After being processed by the data preprocessing module, the dataset is divided into a training set and a test set. A subset containing only minority class samples is extracted from the training set and fed into the data augmentation module. This module first uses the VAE-WACGAN model to learn the data distribution characteristics of these minority class samples. Then, new minority class samples are generated based on the features learned by the model. Finally, these newly generated minority class samples are added to the original training set to construct a balanced dataset with equal class distribution.

#### 4.2.3. Classification Module

The classification module is responsible for training and testing the classification model. Firstly, it trains the classification model using the balanced dataset. Then, the model’s performance is evaluated using the test set. 

## 5. Experiment and Analysis

In this section, the effectiveness of the VAE-WACGAN method is validated. Firstly, the dataset used for the experiments is introduced. Secondly, the evaluation metrics employed in the experiments are described. Thirdly, the specific processes of data preprocessing and feature selection are elaborated in detail. Finally, the effectiveness of the VAE-WACGAN method is demonstrated through a series of experiments. Detailed information about the experimental environment is provided in Table 4.

### 5.1. Dataset

The experimental validation utilized two datasets commonly used in the field of intrusion detection: UNSW-NB15 [37] and CIC-IDS2017 [9].

The UNSW-NB15 dataset consists of 43 feature attributes and two label attributes, encompassing samples of one normal type and nine attack types. The training set contains 175,341 samples, while the test set contains 82,332 samples. The detailed number of samples for each class is shown in Table 5.

The CIC-IDS2017 dataset encompasses five days of network traffic data, with the first day featuring only normal traffic and the subsequent four days including both normal and various types of attack data. The dataset contains 78 feature attributes and one label attribute. Due to experimental setup constraints, 10% of the samples were randomly extracted from the complete dataset to form an experimental subset. Categories with insufficient samples were removed to ensure data quality and reduce the impact of sparse categories. The final sample distribution of the CIC-IDS2017 dataset used for the experiments is presented in Table 6.

### 5.2. Evaluation Metrics

The evaluation metrics used in this study include accuracy, precision, recall, false positive rate (FPR), F1 score, and G-means to assess the performance of the model. Accuracy reflects the overall performance of the model. Precision indicates the accuracy of the model in predicting positive classes. Recall measures the model’s ability to detect positive class samples. The F1 score represents the combined performance of precision and recall. The FPR indicates the probability of the model making errors in detecting normal samples. G-means reflects the model’s overall performance under class imbalance conditions. The formulas for these metrics are as follows:

(17) $$\mathrm{Accuracy}=\frac{TP+TN}{TP+TN+FP+FN}$$

(18) $$\mathrm{Precision}=\frac{TP}{TP+FP}$$

(19) $$\mathrm{Recall}=\frac{TP}{TP+FN}$$

(20) $$F_{1}=2\times \frac{\mathrm{Precision}\times \mathrm{Recall}}{\mathrm{Precision}+\mathrm{Recall}}=\frac{2\times TP}{2\times TP+FP+FN}$$

(21) $$\mathrm{FPR}=\frac{FP}{FP+TN}$$

(22) $$\text{G-means}=\sqrt{\left(\frac{TP}{TP+FN}\right)\times \left(\frac{TN}{TN+FP}\right)}$$

where TP, TN, FP, and FN denote the number of true positives, true negatives, false positives, and false negatives, respectively.

### 5.3. Data Preprocessing and Feature Selection

As described in Section 4.2.1, the raw datasets are first subjected to preprocessing. This process primarily involves two key steps: data preprocessing and feature selection.

(1) Data preprocessing

Data cleaning: For the UNSW-NB15 dataset, while there are no infinite values, there are a significant number of missing values, especially in the ‘service’ column, which contains 141,321 missing entries. Over half of the samples have missing values, and removing these would lead to a substantial loss of information. Therefore, another method was used for handling missing values: filling them with the mode of the ‘service’ column for each category. For the CIC-IDS2017 dataset, given its large number of samples and the relatively few occurrences of missing and infinite values, these samples were directly removed.

Feature encoding: For the UNSW-NB15 dataset, there are three categorical feature columns: “proto”, “service”, and “state”. The one-hot encoding method was used to convert these categorical features. After this process, the feature dimension of the samples increased to 195 dimensions. Additionally, the sample labels in the UNSW-NB15 dataset encompass 10 categories. Label Encoding was applied to transform these categories into integer values ranging from 0 to 9. For the CIC-IDS2017 dataset, since it does not contain any categorical feature columns, only the sample labels needed to be processed. This dataset includes nine categories of sample labels, and Label Encoding was used to map each label to a unique integer value.

Normalization: The feature columns of both datasets were normalized using the min-max normalization method.

(2) Feature selection

**Figure 5:**
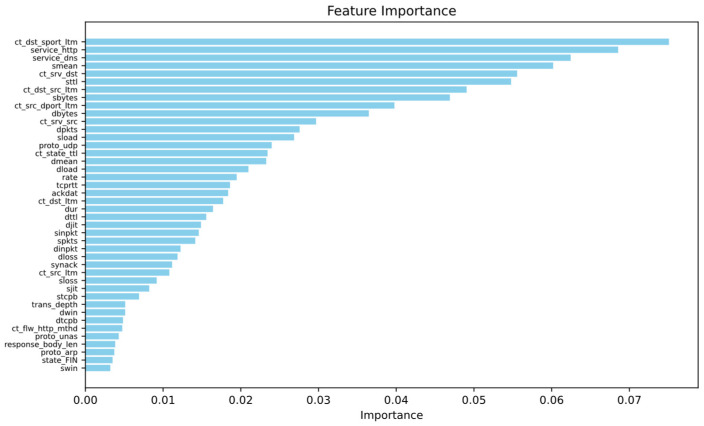
Features with importance scores exceeding 0.003.

**Figure 6:**
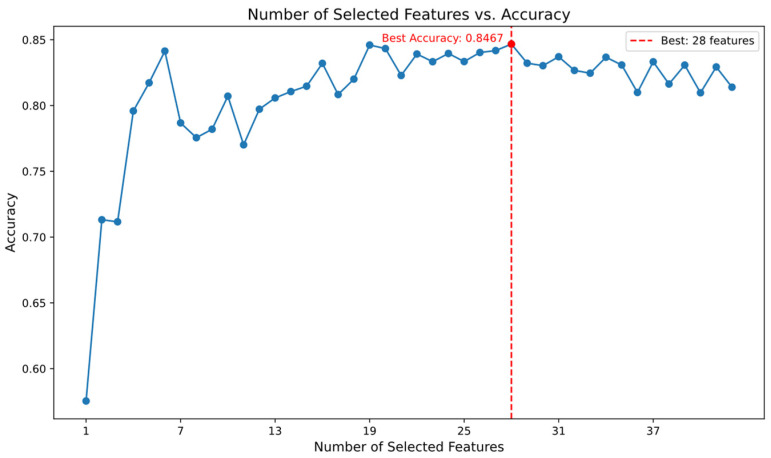
Classification accuracy of MLP for different numbers of features.

After preprocessing, the UNSW-NB15 dataset had a high dimensionality of 195 features. Given the potential negative impact of high-dimensional sparsity on model performance, the Random Forest algorithm was employed for feature selection. Features with an importance score above 0.003 were initially selected. The optimal number of features was subsequently determined by evaluating the classification accuracy of various feature subsets using an MLP classifier. Figure 5 displays the features with importance scores exceeding 0.003, while Figure 6 illustrates the classification accuracy of the MLP classifier for different numbers of features. As a result, the number of features in the dataset was reduced to 28 dimensions.

The CIC-IDS2017 dataset did not undergo one-hot encoding and therefore had lower data sparsity, so no feature selection was performed.

### 5.4. Experimental Procedure and Results Analysis

#### 5.4.1. The Training Experiment of the VAE-WACGAN Model

As described in Section 4.2.2, the dataset was divided into a training set and a test set after preprocessing. A subset containing only minority class samples was selected from the training set for training the VAE-WACGAN model. For the UNSW-NB15 dataset, these minority classes include Analysis, Backdoor, Shellcode, and Worms. For the CIC-IDS2017 dataset, the minority classes include DoS GoldenEye, FTP-Patator, SSH-Patator, DoS slowloris, and DoS Slowhttptest.

The hyperparameters for training the VAE-WACGAN model were determined through extensive experimentation. The model is set to train for 2000 epochs; each batch consists of 256 samples; the learning rates for the encoder, decoder, and discriminator are set to 0.001, 0.001, and 0.0001, respectively. Before each training iteration of the encoder and decoder, the discriminator is trained five times; the weight for the reconstruction loss is set to 1.

**Figure 7:**
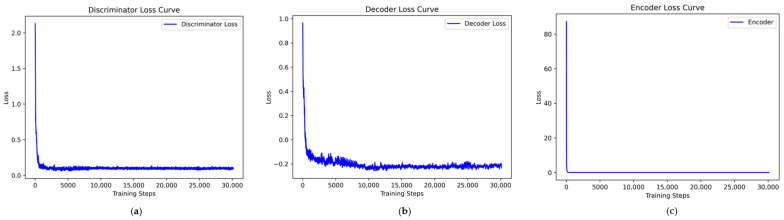
VAE-WACGAN loss curves: (**a**) discriminator loss curve; (**b**) decoder loss curve; (**c**) encoder loss curve.

**Figure 8:**
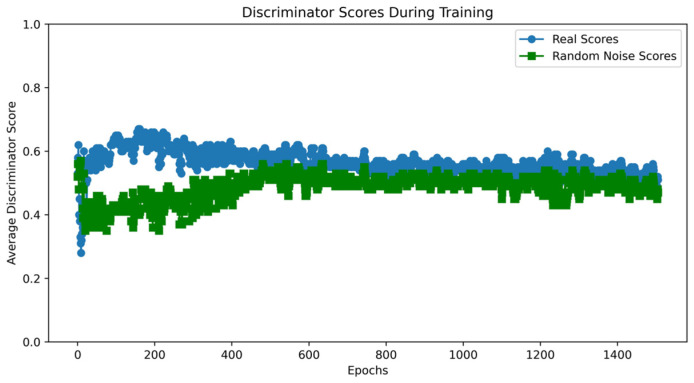
VAE-WACGAN discriminator score variation.

Taking the UNSW-NB15 dataset as an example, the VAE-WACGAN model was trained, and the loss curves and discriminator score variation during the training process were plotted. As shown in Figure 7, the losses of the discriminator, decoder, and encoder all rapidly decrease at the beginning of the training and converge after approximately 10,000 batch iterations. This indicates effective learning in both the generative and discriminative parts of the model during adversarial training. According to Figure 8, after 1000 training epochs, the discriminator scores for both real and fake samples converge to approximately 0.5, indicating that the VAE-WACGAN model has reached an equilibrium state and achieved convergence. At this point, the fake samples generated by the decoder are highly similar to real samples, making it difficult for the discriminator to distinguish between them.

#### 5.4.2. The Training Experiment of the VAEGAN Model

The VAE-WACGAN model is an improved version of the VAEGAN model. To validate the effectiveness of the VAE-WACGAN model, a longitudinal comparison of the two models is necessary. 

A training experiment for the VAEGAN model was conducted. Its network structure is shown in Table 7, with the symbols having the same meanings as described in Section 4.1.1. The training hyperparameters for VAEGAN were determined through multiple experiments, including setting the number of training epochs to 1000, training each batch with 256 samples, and setting the learning rates for the encoder, decoder, and discriminator to 0.000521, 0.000521, and 0.0001, respectively. The weight for the reconstruction loss is set to 1. The configuration of the loss functions and the detailed training process can be referenced from the original VAEGAN paper [5].

**Figure 9:**
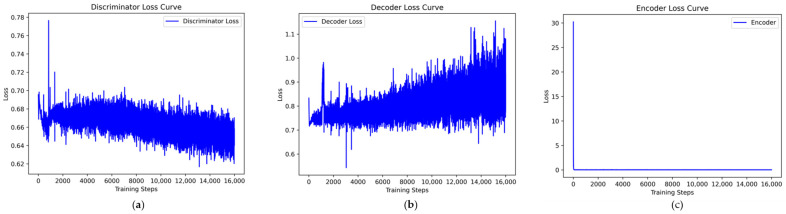
VAEGAN loss curves: (**a**) discriminator loss curve; (**b**) decoder loss curve; (**c**) encoder loss curve.

**Figure 10:**
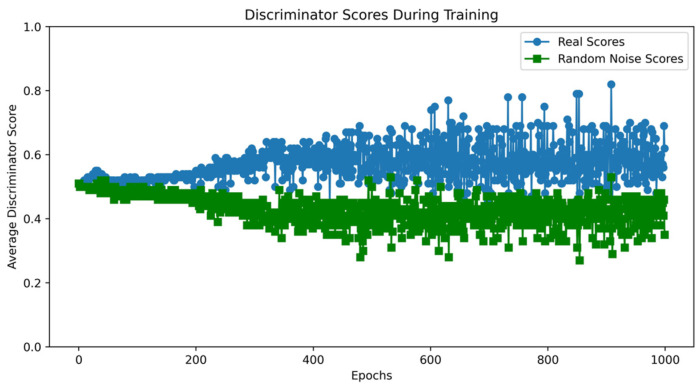
VAEGAN discriminator score variation.

Since the VAEGAN model cannot simultaneously learn the data distribution of multiple minority class samples, a separate learning strategy is employed. Taking the Analysis class data from the UNSW-NB15 dataset as an example, the VAEGAN model was trained, and the loss curves and discriminator score variations during the training process were plotted. According to Figure 9, although the discriminator and decoder losses of the VAEGAN model show a general downward trend during training, they fluctuate significantly, reflecting instability and difficulty in effective convergence during the training process. In contrast, the losses of the discriminator and decoder in the VAE-WACGAN model steadily decrease and quickly stabilize during training, indicating that the VAE-WACGAN model demonstrates significantly better stability during the training process compared to the VAEGAN model. Comparing Figure 8 and Figure 10, it can be seen that the discriminator scores for both real and fake samples tend towards 0.5 at the end of training for both the VAE-WACGAN and VAEGAN models. However, the VAE-WACGAN model exhibits smaller fluctuations in scores, indicating that the VAE-WACGAN model achieves better final convergence.

#### 5.4.3. Comparison of the VAE-WACGAN with Various Class-Balancing Methods

To objectively evaluate the data augmentation effectiveness of the VAE-WACGAN algorithm, it is compared with three traditional class-balancing algorithms: ROS, SMOTE, and ADASYN, as well as the VAEGAN algorithm.

The performance of different class-balancing algorithms was validated using an MLP [38] classification model. This classification model was obtained through the scikit-learn (sklearn) library and configured with specific parameters, including the use of the Adam optimizer, a regularization parameter of 1 × 10^−4^, three hidden layers of sizes 256, 128, and 64, and a maximum of 200 iterations.

**Figure 11:**
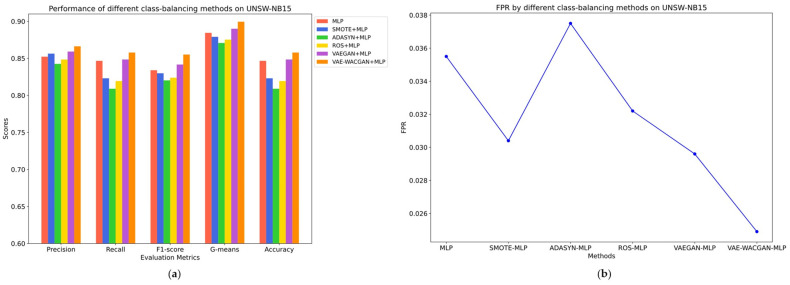
Performance of different class balancing methods on UNSWNB15: (**a**) Precision, Recall, F1-Score, G-means, and Accuracy of different class balancing methods on UNSW-NB15; (**b**) FPR of different class balancing methods on UNSW-NB15.

**Figure 12:**
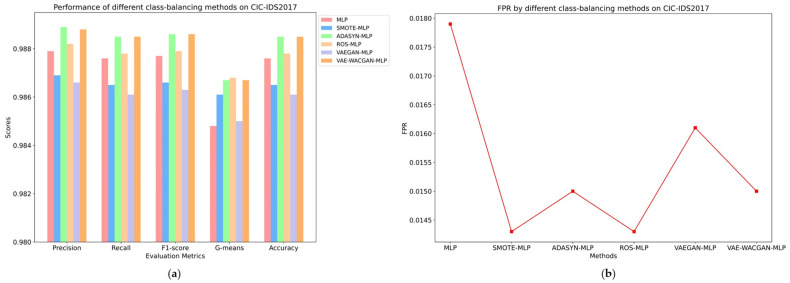
Performance of different class-balancing methods on CIC-IDS2017: (**a**) Precision, Recall, F1-Score, G-means, and Accuracy of different class-balancing methods on UNSW-NB15; (**b**) FPR of different class-balancing methods on CIC-IDS2017.

The classes and the number of samples generated by each class-balancing method were consistent, as shown in Table 8 and Table 9. The performance of different class-balancing methods on the UNSW-NB15 dataset is illustrated in Figure 11 and Table 10, while the performance on the CIC-IDS2017 dataset is shown in Figure 12 and Table 11.

On the UNSW-NB15 dataset, traditional class-balancing methods such as SMOTE, ROS, and ADASYN negatively impact the classifier’s performance. 

Specifically, SMOTE reduces the false positive rate by 0.51% and improves precision by 0.41%. However, it results in decreased recall by 2.36%, F1 score by 0.41%, G-means by 0.54%, and accuracy by 2.36%. Similarly, ROS lowers the false positive rate by 0.33%, but it results in a decrease in precision by 0.38%, recall by 2.73%, F1 score by 1.01%, G-means by 0.92%, and accuracy by 2.73%. Meanwhile, ADASYN leads to declines in all metrics. These negative impacts suggest that traditional class-balancing methods are insufficient for capturing the complex data distribution, thereby introducing noise points that do not conform to the true distribution characteristics, ultimately reducing the classifier’s performance.

In contrast, the VAEGAN method enhances performance, with precision increasing by 0.69%, recall by 0.18%, F1 score by 0.77%, G-means by 0.55%, and accuracy by 0.18%, alongside a decrease in the false positive rate by 0.59%. And the VAE-WACGAN method provides the most significant improvement, increasing precision by 1.40%, recall by 1.12%, F1 score by 2.12%, G-means by 1.5%, and accuracy by 1.12%, while also decreasing the false positive rate by 1.06%. This indicates that both the VAEGAN and VAE-WACGAN methods effectively capture the distribution characteristics of the UNSW-NB15 dataset, resulting in the generation of high-quality samples and enhanced classifier performance. Nonetheless, based on the final classifier metrics, the VAE-WACGAN method exhibits superior data augmentation effects compared to the VAEGAN method.

On the CIC-IDS2017 dataset, SMOTE and VAEGAN algorithms negatively impact the classifier’s performance, while ROS, ADASYN, and VAE-WACGAN algorithms effectively enhance the classifier’s performance. 

Although the SMOTE and VAEGAN algorithms improve the classifier’s G-means and false positive rate (SMOTE increases the G-means by 0.13% and reduces the false positive rate by 0.36%; VAEGAN increases the G-means by 0.02% and reduces the false positive rate by 0.018%), the remaining four metrics all decline. This indicates that these two algorithms do not effectively capture the distribution characteristics of the CIC-IDS2017 dataset, thereby introducing noise data. 

For the ROS, ADASYN, and VAE-WACGAN algorithms, all performance metrics of the classifier are effectively improved. 

Specifically, the ROS algorithm increases the classifier’s precision by 0.03%, recall by 0.02%, F1 score by 0.02%, reduces the false positive rate by 0.36%, increases G-means by 0.2%, and improves accuracy by 0.02%. 

The ADASYN and VAE-WACGAN algorithms both improve the classifier’s precision by approximately 0.1% (ADASYN by 0.1%, VAE-WACGAN by 0.09%), recall by 0.09%, F1 score by 0.09%, reduce the false positive rate by 0.29%, increase G-means by 0.19%, and improve accuracy by 0.09%. This indicates that these three algorithms effectively capture the distribution characteristics of the CIC-IDS2017 dataset, thereby generating high-quality samples. Regarding various performance metrics, the enhancement effect of the VAE-WACGAN algorithm is comparable to ADASYN and superior to the other class-balancing methods.

The above experimental results indicate that on both the UNSW-NB15 and CIC-IDS2017 datasets, the VAE-WACGAN method effectively improves all performance metrics of the classifier and has superior data augmentation effects compared to the other four class-balancing methods. However, VAE-WACGAN shows a lower performance than ADASYN on the CIC-IDS2017 dataset. This is likely due to CIC-IDS2017’s simpler characteristics, which make it easier to classify. Consequently, simpler methods like ADASYN prove more effective in addressing class imbalance, whereas the more sophisticated VAE-WACGAN method may not perform as well in this less complex context.

#### 5.4.4. Analysis of Model Complexity

Analyzing model complexity is essential for understanding the computational requirements and efficiency of a model. In this experiment, a comparative complexity analysis of VAE-WACGAN and VAEGAN was conducted on two datasets to rigorously evaluate the performance of VAE-WACGAN and validate its improvements.

Model complexity is typically measured using two key metrics: Floating Point Operations (FLOPs) and the number of parameters. FLOPs represent the total number of floating point operations required for a single forward pass, reflecting the model’s time complexity. The number of parameters refers to the total count of trainable parameters, indicating the model’s space complexity. The complexity of the VAE-WACGAN and VAEGAN models across two datasets is presented in Table 12.

Table 12 reveals that the VAE-WACGAN model exhibits markedly higher complexity compared to VAEGAN across both datasets. Specifically, on the UNSW-NB15 dataset, VAE-WACGAN has 2.743 GFLOPs and 1.509 million parameters, significantly exceeding VAEGAN’s 0.331 GFLOPs and 0.738 million parameters. Similarly, on the CIC-IDS2017 dataset, VAE-WACGAN’s complexity is 3.732 GFLOPs and 4.379 million parameters, which is considerably higher than VAEGAN’s 0.976 GFLOPs and 2.072 million parameters. These results indicate that while VAE-WACGAN demands greater computational resources and incurs higher storage costs than VAEGAN, it delivers superior data augmentation performance on both datasets, as demonstrated in Table 10 and Table 11. The increased complexity of VAE-WACGAN enhances its representational capability, allowing it to capture more intricate data features and thereby improve its effectiveness in data augmentation tasks.

#### 5.4.5. Visualization Comparison of the Original and Balanced Datasets

**Figure 13:**
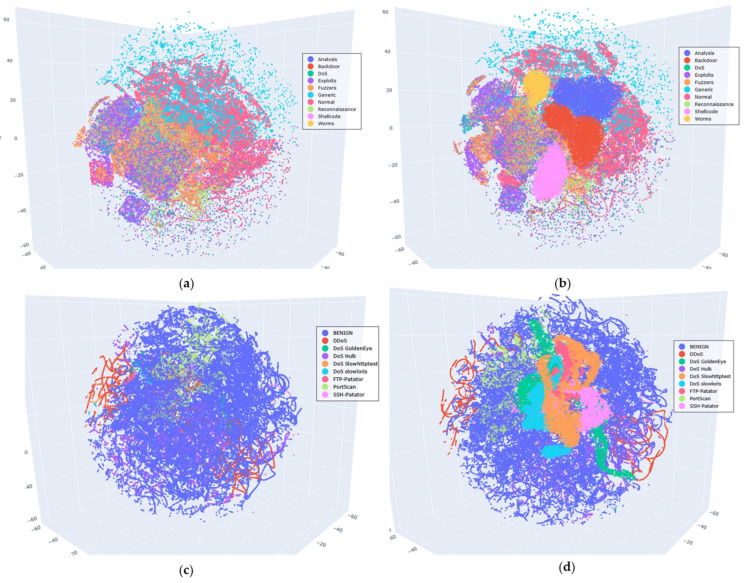
Visualization of the original and balanced datasets: (**a**) visualization of the UNSW-NB15 original dataset; (**b**) visualization of the UNSW-NB15 balanced dataset; (**c**) visualization of the CIC-IDS2017 original dataset; (**d**) visualization of the CIC-IDS2017 balanced dataset.

In the previous analysis, the effectiveness of the VAE-WACGAN method was validated from three dimensions: the training process curves, the performance comparison of class-balancing methods, and model complexity. To more intuitively assess the data augmentation effect of the VAE-WACGAN method, this experiment visualizes the comparison between the original dataset and the balanced dataset processed by the VAE-WACGAN method. The steps were as follows: the t-SNE (t-Distributed Stochastic Neighbor Embedding) algorithm [39] was first used to perform dimensionality reduction on the original and balanced datasets, and then both were visualized. Given the large sample size and numerous categories of the datasets used, reducing the data to two dimensions may not clearly show the distribution characteristics. Therefore, the data was reduced to three dimensions for visualization. The visualization results are shown in Figure 13. 

Figure 13a,b reveals that the original UNSW-NB15 dataset exhibits severe class imbalance, with samples from minority classes such as Analysis, Backdoor, Shellcode, and Worms being so scarce that they are nearly indistinguishable in (a). However, after applying the VAE-WACGAN method, these minority class samples become visible in (b), with a notable increase in their proportions. As shown in Figure 13c,d, the original CIC-IDS2017 dataset also has a class imbalance issue. However, after processing with the VAE-WACGAN method, the minority classes are significantly enhanced in the balanced dataset.

These experimental results indicate that, from the intuitive visualization, the VAE-WACGAN method can increase the proportion of minority class samples, effectively addressing the class imbalance issue in the datasets.

#### 5.4.6. Multi-Classifier Validation of the VAE-WACGAN Data Augmentation

**Figure 14:**
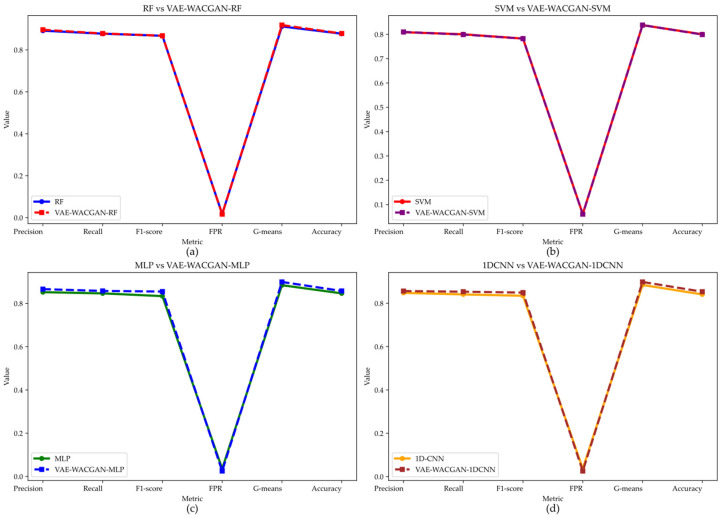
Performance of the VAE-WACGAN method on different classifiers for the UNSW-NB15 dataset. (**a**) Performance of the VAE-WACGAN method on the RF classifier; (**b**) Performance of the VAE-WACGAN method on the SVM classifier; (**c**) Performance of the VAE-WACGAN method on the MLP classifier; (**d**) Performance of the VAE-WACGAN method on the 1DCNN classifier.

**Figure 15:**
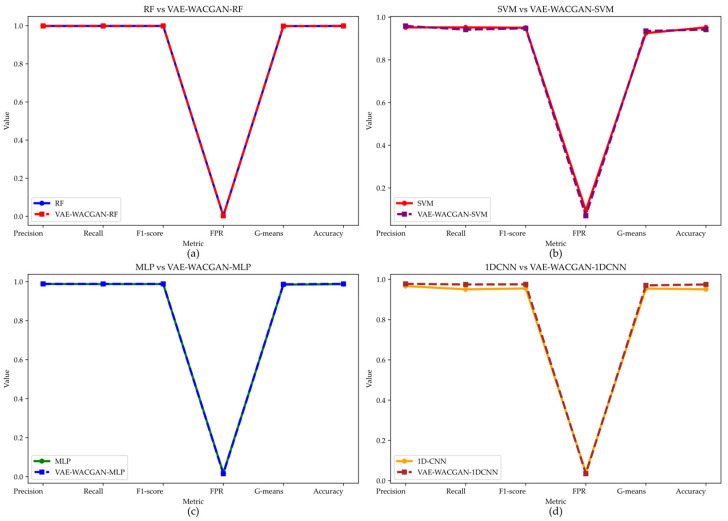
Performance of the VAE-WACGAN method on different classifiers for the CIC-IDS2017 dataset. (**a**) Performance of the VAE-WACGAN method on the RF classifier; (**b**) Performance of the VAE-WACGAN method on the SVM classifier; (**c**) Performance of the VAE-WACGAN method on the MLP classifier; (**d**) Performance of the VAE-WACGAN method on the 1DCNN classifier.

To comprehensively evaluate the VAE-WACGAN method, this experiment employs four different classifiers to observe the performance changes after applying the VAE-WACGAN method. The four classifiers include two shallow classifiers: Random Forest (RF) [40] and Support Vector Machine (SVM) [41]; and two deep classifiers: Multi-Layer Perceptron (MLP) [38] and One-Dimensional Convolutional Neural Network (1DCNN) [42]. The performance of each classifier on the UNSW-NB15 dataset is shown in Figure 14 and Table 13, and on the CIC-IDS2017 dataset in Figure 15 and Table 14.

The 1DCNN classifier consists of a four-layer network structure. The first two layers are convolutional layers, followed by two fully connected layers. Each of the first three layers applies batch normalization and the ReLU activation function. Both convolutional layers have a kernel size of 3, with stride and padding set to 1, and the number of kernels set to 32 and 64, respectively. The first fully connected layer contains 256 neurons, while the second fully connected layer outputs classification predictions, with the number of neurons corresponding to the number of sample classes. The following training hyperparameters were set to optimize the 1DCNN: a total of 200 training epochs, a batch size of 512 samples per epoch, a learning rate of 0.001, and the Adam optimizer for weight updates. These hyperparameters were determined through multiple experiments.

On the UNSW-NB15 dataset, the VAE-WACGAN method significantly enhances the performance of most classifiers, especially for the MLP and 1DCNN classifiers. 

Specifically, the VAE-WACGAN method notably improves the MLP and 1DCNN classifiers. For the MLP classifier, VAE-WACGAN-MLP improves Precision from 0.8523 to 0.8663, Recall from 0.8467 to 0.8579, F1 score from 0.8340 to 0.8552, G-means from 0.8845 to 0.8995, Accuracy from 0.8467 to 0.8579, and reduces FPR from 0.0355 to 0.0249. For the 1DCNN classifier, VAE-WACGAN-1DCNN improves Precision from 0.848 to 0.8563, Recall from 0.8408 to 0.8533, F1 score from 0.8345 to 0.8492, G-means from 0.8845 to 0.8983, Accuracy from 0.8408 to 0.8533, and reduces FPR from 0.0364 to 0.0256.

The VAE-WACGAN method slightly improves the performance of the RF classifier. Notably, VAE-WACGAN-RF improves Precision from 0.8914 to 0.8960, Recall from 0.8771 to 0.8781, F1 score from 0.8673 to 0.8674, G-means from 0.9116 to 0.9182, Accuracy from 0.8771 to 0.8781, and reduces FPR from 0.0173 to 0.0167.

For the SVM classifier, VAE-WACGAN-SVM performs excellently in Precision, FPR, and G-means, with Precision increasing from 0.8088 to 0.8099, FPR decreasing from 0.0615 to 0.0606, and G-means increasing from 0.8379 to 0.8381. However, there is a slight decrease in Recall, F1 score, and Accuracy, leading to a minor overall decline in performance.

On the CIC-IDS2017 dataset, the VAE-WACGAN method also enhances classifier performance. 

For the MLP and 1DCNN classifiers, the VAE-WACGAN method improves all evaluation metrics. Compared to MLP, VAE-WACGAN-MLP increases Precision from 0.9879 to 0.9888, Recall from 0.9876 to 0.9885, F1 score from 0.9877 to 0.9886, G-means from 0.9848 to 0.9867, Accuracy from 0.9876 to 0.9885, and reduces FPR from 0.0179 to 0.015. Compared to 1DCNN, VAE-WACGAN-1DCNN increases Precision from 0.9666 to 0.9776, Recall from 0.9505 to 0.9746, F1-score from 0.9547 to 0.9754, G-means from 0.9542 to 0.9699, Accuracy from 0.9505 to 0.9746, and reduces FPR from 0.0415 to 0.0345. 

For the RF classifier, VAE-WACGAN-RF improves Precision from 0.9986 to 0.9987, Recall from 0.9986 to 0.9987, F1 score from 0.9986 to 0.9987, G-means from 0.9978 to 0.9979, Accuracy from 0.9986 to 0.9987, and reduces FPR from 0.003 to 0.0029. These results indicate that the VAE-WACGAN method marginally improves the performance of the RF classifier, positively impacting key metrics.

For the SVM classifier, although VAE-WACGAN-SVM improves Precision and FPR, with Precision increasing from 0.9521 to 0.9589 and FPR decreasing from 0.0944 to 0.0695, Recall, F1-score, G-means, and Accuracy all decline, resulting in an overall performance drop.

In summary, the VAE-WACGAN method demonstrates significant data augmentation effects across different classifiers, especially for the MLP and 1DCNN classifiers, further validating the superior data augmentation capability of the VAE-WACGAN method.

#### 5.4.7. Comparison with Recent Advanced Methods

The multi-classifier validation experiment in Section 5.4.6 indicated that the intrusion detection model achieved optimal performance with the RF classifier. Therefore, this experiment compares the VAE-WACGAN-RF model with recent advanced approaches to assess the feasibility of the proposed intrusion detection method, as shown in Table 15 and Table 16.

Based on Table 15 and Table 16, our method consistently outperforms the other three advanced intrusion detection approaches across all evaluation metrics on both the UNSW-NB15 and CIC-IDS2017 datasets. Notably, in terms of the F1-score—a critical metric that balances precision and recall—our method demonstrates substantial improvements. On the UNSW-NB15 dataset, our method exceeds FCWGAN-BiLSTM by 0.9%, CNN-BiLSTM by 6.79%, and MCNN-DFS by 5.74%. Similarly, on the CIC-IDS2017 dataset, our approach outperforms KD-TCNN by 0.41%, KNN-TACGAN by 4.06%, and GAN-RF by 4.83%. These results clearly demonstrate the effectiveness of our method in performing intrusion detection.

## 6. Conclusions and Future Work

To address the class imbalance issue in network intrusion detection, a novel generative adversarial network model named VAE-WACGAN is proposed, which generates samples of minority classes to balance the dataset. The effectiveness of the VAE-WACGAN method has been validated through multiple experiments. Experimental results indicate that the VAE-WACGAN model is stable and easy to converge during training. Compared to class-balancing methods such as ROS, SMOTE, ADASYN, and VAEGAN, the VAE-WACGAN method can accurately capture the distribution characteristics of complex data, thereby generating samples more similar to the original data and effectively addressing the class imbalance issue in network intrusion detection. Additionally, the intrusion detection method based on VAE-WACGAN was compared with other advanced techniques, demonstrating superior performance across various metrics.

Data insufficiency is not only a challenge in intrusion detection tasks but is also a widespread issue across many machine learning domains. In the future, we plan to extend our approach to the field of data augmentation by utilizing the VAE-WACGAN model proposed in this paper to generate high-quality synthetic data. Data augmentation techniques expand datasets by generating new training samples, thereby enhancing the model’s generalization capabilities and robustness. This method effectively addresses the problem of data scarcity, improving model performance and stability across various applications. In particular, in fields where data are limited or costly to obtain, such as medical imaging analysis, synthetic data generation not only enriches training datasets but also reduces reliance on real-world data, driving progress in these applications.

## Figures and Tables

**Table 1:** Network structure of Encoder.

Model	Layer	Filters	Kernel Size	Output Shape	Activation
Encoder	Embedding	-	-	feature_num	-
	Conv1d	32	3	(32, feature_num − 2)	ReLU
	Conv1d	64	3	(64, feature_num − 4)	ReLU
	Flatten	-	-	64 × (feature_num − 4)	-
	Linear	-	-	256	-
	Linear	-	-	128	-
	Linear	-	-	128	-

**Table 2:** Network structure of Decoder.

Model	Layer	Filters	Kernel Size	Output Shape	Activation
Decoder	Embedding	-	-	feature_num	-
	Linear	-	-	64 × (feature_num − 4)	ReLU
	Unflatten	-	-	(64, feature_num − 4)	-
	ConvTranspose1d	64	3	(64, feature_num − 2)	ReLU
	ConvTranspose1d	32	3	(32, feature_num)	ReLU
	ConvTranspose1d	1	1	(1, feature_num)	ReLU

**Table 3:** Network structure of Discriminator.

Model	Layer	Filters	Kernel Size	Output Shape	Activation
Discriminator	Conv1d	32	3	(32, feature_num − 2)	ReLU
	Conv1d	64	3	(64, feature_num − 4)	ReLU
	Flatten	-	-	64 × (feature_num − 4)	-
	Linear	-	-	512	-
	Linear	-	-	64	ReLU
	Linear (adversarial part)	-	-	1	Sigmoid
	Linear (classification part)	-	-	classes	-

**Table 4:** Experimental environment configuration.

Experimental Platform	Configuration
Operating System	Windows 11 22H2
CPU	AMD Ryzen 9 7945HX with Radeon Graphics
GPU	NVIDIA GeForce RTX 4060 Laptop
Memory	16.0GB
IDE Tool	PyCharm Community Edition 2020.1.3 x64
Programming Language	Python3.9
Deep Learning Framework	PyTorch 1.12.0 + cu113, torchvision 0. 13. 0 + cu113

**Table 5:** Sample distribution of UNSW-NB15.

Type	Training Set	Testing Set
Normal	56,000	37,000
Generic	40,000	18,871
Exploits	33,393	11,132
Fuzzers	18,184	6062
Dos	12,264	4089
Reconnaissance	10,491	3496
Analysis	2000	677
Backdoor	1746	583
Shellcode	1133	378
Worms	130	44
Sum	175,341	82,332

**Table 6:** Sample distribution of CIC-IDS2017.

Type	Training Set	Testing Set
BENIGN	180,747	45,246
DoS Hulk	18,388	4645
PortScan	12,510	3099
DDoS	10,234	2484
DoS GoldenEye	906	218
FTP-Patator	591	158
SSH-Patator	470	119
DoS slowloris	461	114
DoS Slowhttptest	460	109
Sum	224,767	56,192

**Table 7:** The network structure of VAEGAN.

Model	Layer	Filters	Kernel Size	Output Shape	Activation
Encoder	Conv1d	32	3	(32, feature_num − 2)	ReLU
	Conv1d	64	3	(64, feature_num − 4)	ReLU
	Flatten	-	-	64 × (feature_num − 4)	-
	Linear	-	-	256	-
	Linear	-	-	128	-
	Linear	-	-	128	-
Decoder	Linear	-	-	64 × (feature_num − 4)	ReLU
	Unflatten	-	-	(64, feature_num − 4)	-
	ConvTranspose1d	64	3	(64, feature_num − 2)	ReLU
	ConvTranspose1d	32	3	(32, feature_num)	ReLU
	ConvTranspose1d	1	1	(1, feature_num)	ReLU
Discriminator	Conv1d	32	3	(32, feature_num − 2)	ReLU
	Conv1d	64	3	(64, feature_num − 4)	ReLU
	Flatten	-	-	64 × (feature_num − 4)	-
	Linear	-	-	32	ReLU
	Linear	-	-	1	Sigmoid

**Table 8:** The generated samples for the UNSWNB15 dataset.

Class	Number of Original Samples	Number of Generated Samples	Sum
Normal	56,000	0	56,000
Generic	40,000	0	40,000
Exploits	33,393	0	33,393
Fuzzers	18,184	0	18,184
Dos	12,264	0	12,264
Reconnaissance	10,491	0	10,491
Analysis	2000	10,000	12,000
Backdoor	1746	10,000	11,746
Shellcode	1133	10,000	11,133
Worms	130	10,000	10,130
Sum	175,341	40,000	215,341

**Table 9:** The generated samples for the CIC-IDS2017 dataset.

Class	Number of Original Samples	Number of Generated Samples	Sum
BENIGN	180,747	0	180,747
DoS Hulk	18,388	0	18,388
PortScan	12,510	0	12,510
DDoS	10,234	0	10,234
DoS GoldenEye	906	10,000	10,906
FTP-Patator	591	10,000	10,591
SSH-Patator	470	10,000	10,470
DoS slowloris	461	10,000	10,461
DoS Slowhttptest	460	10,000	10,460
Sum	224,767	50,000	274,767

**Table 10:** Performance of different class-balancing methods on UNSW-NB15.

Methods	Precision	Recall	F1-Score	FPR	G-Means	Accuracy
MLP	0.8523	0.8467	0.8340	0.0355	0.8845	0.8467
SMOTE-MLP	0.8564	0.8231	0.8299	0.0304	0.8791	0.8231
ADASYN-MLP	0.8426	0.8090	0.8203	0.0375	0.8708	0.8090
ROS-MLP	0.8485	0.8194	0.8239	0.0322	0.8753	0.8194
VAEGAN-MLP	0.8592	0.8485	0.8417	0.0296	0.89	0.8485
**VAE-WACGAN-MLP**	**0.8** **663**	**0.8579**	**0.85** **52**	**0.0249**	**0.8995**	**0.8579**

**Table 11:** Performance of different class-balancing methods on CIC-IDS2017.

Methods	Precision	Recall	F1-Score	FPR	G-Means	Accuracy
MLP	0.9879	0.9876	0.9877	0.0179	0.9848	0.9876
SMOTE-MLP	0.9869	0.9865	0.9866	0.0143	0.9861	0.9865
ADASYN-MLP	0.9889	0.9885	0.9886	0.015	0.9867	0.9885
ROS-MLP	0.9882	0.9878	0.9879	0.0143	0.9868	0.9878
VAEGAN-MLP	0.9866	0.9861	0.9863	0.0161	0.985	0.9861
**VAE-WACGAN-MLP**	**0.9888**	**0.9885**	**0.9886**	**0.015**	**0.9867**	**0.9885**

**Table 12:** Model Complexity for VAE-WACGAN and VAEGAN on Two Datasets.

Dataset	Model	FLOPs (G)	Parameters (M)
UNSW-NB15	VAE-WACGAN	2.743	1.509
	VAEGAN	0.331	0.738
CIC-IDS2017	VAE-WACGAN	3.732	4.379
	VAEGAN	0.976	2.072

**Table 13:** Performance of different classification models on the UNSW-NB15 dataset.

Classifier	Precision	Recall	F1-Score	FPR	G-Means	Accuracy
RF	0.8914	0.8771	0.8673	0.0173	0.9116	0.8771
**VAE-WACGAN-RF**	**0.89** **60**	**0.8781**	**0.86** **74**	**0.01** **67**	**0.9** **182**	**0.8** **781**
SVM	0.8088	0.8003	0.7826	0.0615	0.8379	0.8003
VAE-WACGAN-SVM	0.8099	0.7991	0.7824	0.0606	0.8381	0.7991
MLP	0.8523	0.8467	0.8340	0.0355	0.8845	0.8467
**VAE-WACGAN-MLP**	**0.8** **6** **63**	**0.8579**	**0.8** **552**	**0.02** **49**	**0.89** **95**	**0.8** **579**
1D-CNN	0.848	0.8408	0.8345	0.0364	0.8845	0.8408
**VAE-WACGAN-1DCNN**	**0.8** **563**	**0.8533**	**0.8** **492**	**0.0** **256**	**0.8** **983**	**0.8** **533**

**Table 14:** Performance of different classification models on the CIC-IDS2017 dataset.

Classifier	Precision	Recall	F1-Score	FPR	G-Means	Accuracy
RF	0.9986	0.9986	0.9986	0.003	0.9978	0.9986
**VAE-WACGAN-RF**	**0.998** **7**	**0.998** **7**	**0.998** **7**	**0.00** **29**	**0.997** **9**	**0.998** **7**
SVM	0.9521	0.9522	0.9505	0.0944	0.9251	0.9522
VAE-WACGAN-SVM	0.9589	0.9415	0.9477	0.0695	0.9355	0.9415
MLP	0.9879	0.9876	0.9877	0.0179	0.9848	0.9876
**VAE-WACGAN-MLP**	**0.9888**	**0.9885**	**0.9886**	**0.015**	**0.9867**	**0.9885**
1DCNN	0.9666	0.9505	0.9547	0.0415	0.9542	0.9505
**VAE-WACGAN-1DCNN**	**0.9776**	**0.9746**	**0.9754**	**0.0345**	**0.9699**	**0.9746**

**Table 15:** Performance comparison of different intrusion detection methods on the UNSW-NB15 dataset.

Model	Precision	Recall	F1-Score	Accuracy
FCWGAN-BiLSTM [43]	0.8611	0.8557	0.8584	0.8559
CNN-BiLSTM [44]	0.8148	0.7847	0.7995	0.7682
MCNN-DFS [45]	0.81	0.81	0.81	0.8051
**Ours**	**0.8960**	**0.8781**	**0.8674**	**0.** **8781**

**Table 16:** Performance comparison of different intrusion detection methods on the CIC-IDS2017 dataset.

Model	Precision	Recall	F1-Score	Accuracy
KD-TCNN [46]	0.9948	0.9947	0.9946	0.9944
KNN-TACGAN [28]	0.9685	0.9479	0.9581	0.9586
GAN-RF [47]	0.9868	0.9276	0.9504	0.9983
**Ours**	**0.9** **987**	**0.9** **987**	**0.9** **987**	**0.9** **987**

## Data Availability

UNSW-NB15 dataset: https://research.unsw.edu.au/projects/unsw-nb15-dataset (accessed on 15 March 2024). CIC-IDS2017 dataset: https://www.unb.ca/cic/datasets/ids-2017.html (accessed on 12 June 2024).
